# Aerobic Exercise Associated with Fish Oil Supplementation Decreases C-Reactive Protein and Interleukin-6 in Celiac Disease Patients

**DOI:** 10.1155/2022/3908675

**Published:** 2022-07-21

**Authors:** Allysson Costa, Gleisson A. P. de Brito

**Affiliations:** Laboratory of Physiology and Developmental Biology, Federal University of Latin American Integration—UNILA, Foz do Iguaçu, Paraná, Brazil

## Abstract

**Background:**

Several studies indicate that celiac disease patients present alterations within anthropometric, metabolic, and inflammatory parameters, while physical exercise and fish oil are known to activate modulatory pathways of such parameters.

**Objective:**

To investigate the effects of a 12-week-long protocol of aerobic exercise and its association with fish oil supplementation in nineteen adult celiac disease patients. *Material and Methods*. The celiacs were divided into 2 groups: (A) FOS: supplementation (*n* = 11); and (B) EXE: supplementation and exercise (*n* = 8). The celiac groups were compared to the adult healthy control group (CTR) (*n* 12). Aerobic exercises were performed weekly, in three sessions of 60 minutes each, with a maximal heart rate intensity of 60–70%. The participants received 2 g/day of fish oil, a daily intake of 420 mg of eicosapentaenoic acid, and 230 mg of docosahexaenoic acid. The following measurements were taken in four phases: (A) anthropometry: body mass, height, body mass index, waist-to-hip ratio, fat mass, and fat-free mass; (B) metabolic profile: total cholesterol, triglycerides, HDL, and LDL; and (C) inflammatory profile: C-reactive protein and interleukin-6.

**Results:**

Supplementation associated with aerobic exercise promoted a significant reduction in C-reactive protein (*P* < 0.01) and increased the proportion of individuals in the undetectable range of interleukin-6.

**Conclusions:**

The associated interventions showed a corrective and preventive potential in relation to disorders associated with chronic inflammation; however, the experimental design does not allow us to discriminate between the biological effects that are dependent on the association between interventions and those exclusively dependent on aerobic exercise.

## 1. Introduction

Celiac disease (CD) is a chronic autoimmune illness that affects 0.5% to 1% of the world population [1]. The CD development factors are related to peptides derived from the digestion of gluten proteins, which in association with HLA genes and anti-transglutaminase antibodies (tTG) promote an intense and specific autoimmune response [2].

The inflammatory response causes small bowel lesions with consequent villi atrophies [3], culminating in an unsatisfactory absorption of nutrients, which can result in lower anthropometric values of body mass, body mass index, fat mass, fat-free mass, and bone mineral content and density, even in a gluten-free diet [4]. Additionally, previous research suggested that CD patients show significantly higher levels of cytokines, interleukin-2 (IL-2), interferon-*γ* (IFN-*γ*), IL-4, IL-6, IL8, IL-10, IL-15, and tumor necrosis factor-alpha (TNF-*α*) [5], and C-reactive protein (CRP) [6]. Some studies suggest that higher concentrations of cytokines are also related to diseases frequently associated with CD, like autoimmune thyroid, hepatitis, autoimmune gastritis, osteopenia, and psychiatric conditions [7, 8].

From another perspective, previously reviewed studies showed a correlation between low concentrations of inflammatory markers, cytokines and CRP, with the increased frequency and intensity of physical activities [9, 10], and the ability of different physical exercise protocols to promote modulatory effects in CRP and cytokines levels [11, 12].

Similar to physical exercise, studies have found that *α*-linolenic acid (ALA) can promote anti-inflammatory effects, decreasing CRP and cytokines levels [13, 14]. Many marine plants carry out chain elongation and the additional desaturation of ALA, resulting in derivative acids: eicosapentaenoic acid (EPA) and docosahexaenoic acid (DHA). Large quantities of EPA and DHA are found in some marine fish oils, which is explained by the food chain transfer from plants to fish [15].

Previously discussed points strongly suggest that such interventions could result in beneficial modulations within CD proinflammatory parameters, contributing to a better prognosis, preventing the appearance of associated proinflammatory disorders and diseases, and improving quality of life.

## 2. Material and Methods

### 2.1. Experimental Design

The sample of this study was made up of 19 celiac disease patients, diagnosed by a medical specialist, with 38.67 ± 41.19 months of a gluten free-diet, and 11 healthy people. Inclusion criteria were as follows: diagnosed CD patients with good adherence to a gluten-free diet [16]; CD patients and healthy people without performing regular physical exercise and taking fish oil supplementation in the last 3 months; and absence of chronic diseases, drugs, alcohol, tobacco, and other medicines.

The enrolled volunteers composed the following groups: (A) FOS group—CD patients submitted to 12 weeks of fish oil supplementation, 11 subjects, 10 females and 1 male, 36.5 ± 10.7 years of age; (B) EXE group—CD patients submitted to 12 weeks of fish oil supplementation and aerobic exercise, 8 subjects, 7 females and 1 male, 38.2 ± 8.8 years of age; and (C) CTR group—healthy people control group, without intervention, 12 subjects, 6 females and 6 males, 31.92 ± 4.99 years of age.

FOS, EXE, and CTR were evaluated for 12 weeks regarding the anthropometry, metabolic profile, inflammatory profile, and average calorie intake in four phases: phase 1—before the protocol; phase 2—week 4; phase 3—week 8; and phase 4—at the end of the protocol, after week 12.

This research was approved by the Ethics Committee of the Assis Gurgacz University Center (CEP-FAG), substantiated term number 2,315,783, October 5, 2017, and every volunteer gave informed consent before participating in the study.

### 2.2. Protocols

#### 2.2.1. Aerobic Exercise

The protocol was performed over 3 sessions per week, with each session including 10 minutes of warm-up exercises, 35 minutes of aerobic exercise, with a maximum heart rate intensity of 60–70%, and 15 minutes of flexibility exercises. The intensity was evaluated through digital frequency meters and the Borg Perception Scale.

#### 2.2.2. Fish Oil Supplementation

During the protocol, each subject received 2 g of fish oil for daily intake, through 2 capsules of 1000 mg, resulting in a daily intake of 420 mg of EPA and 230 mg of DHA. The capsules were ingested at two different times of the day, preferably before meals.

A placebo was not provided because (1) there was no contact between groups and (2) there was no suitable placebo available to the experimenters for fish oil supplementation [17–19]. Providing any other oil could cause its incorporation into the cell membranes and increase oil consumption, which has been associated with the inflammatory parameters.

#### 2.2.3. Anthropometry

Anthropometric parameters were evaluated through the following: height with fixed wall stadiometer; body mass (BM) through digital scale; body mass index (BMI) measured through BM by height^2^; fat mass (% FM) and fat-free mass (FFM) through skinfold thickness; and waist-to-hip ratio (WHR) through the waist circumference by hip waist.

#### 2.2.4. Metabolic Profile

Metabolic profile was measured by triglycerides (TAG), total cholesterol (CHOL), HDL, and LDL and analyzed through the colorimetric enzymatic method, with the concentration calculated in mg/dL.

#### 2.2.5. Inflammatory Profile

The inflammatory profile evaluated CRP and IL-6. CRP analysis was performed by ultrasensitive immunoturbidimetry, with concentration calculated in mg/L and having an analytical sensitivity of 0.06 mg/L. IL-6 analysis was performed by electrochemiluminescence, with concentration calculated in pg/ml and having an analytical sensitivity of 1.5 pg/ml.

#### 2.2.6. Average Calorie Intake

To predict the average intake of calories (CAL), proteins (PTN), carbohydrates (CHO), and lipids (LIP), a three-day food recall model was applied [20, 21].

## 3. Statistical Analysis

Statistical analyses and graphs were generated using the GraphPad prism statistical package, version 6.0. Comparisons between the three groups of variables with normal distribution were tested using ANOVA, with Tukey's posttest, while variables without normal distribution were tested using Kruskal-Wallis, with Dunn's posttest. Comparisons between the two groups were performed using the *t*-test for variables with normal distribution and Mann-Whitney *U* test for variables with no normal distribution. Intragroup comparisons of variables with normal distribution were performed using the paired *t*-test, while variables without normal distribution were tested using the Wilcoxon test. Correlation analyses were performed using the Pearson test for normal distribution and the Spearman test for variables with no normal distribution. Confidence intervals of 95% (*P* ≤ 0.05) were considered to determine significance levels.

## 4. Results

### 4.1. Age and Anthropometric Parameters

Results of the intergroup analysis from anthropometric parameters are shown in Table 1. There was no statistical difference between CTR, FOS, and EXE during phases 1 and 4 in BM, BMI, WHR, and %FM. There was a significant difference between CTR, FOS, and EXE in height (phase 1: *P*=0.0011; phase 4: *P*=0.0011), with CTR showing higher values than FOS (phase 1 and phase 4: *P*=0.0061) and EXE (phases 1 and 4: *P*=0.0027). An FFM difference between groups during phase 1 was identified (*P*=0.0221) which was not confirmed in the posttest (FOS versus EXE: *P* ≥ 0.99; FOS versus CTR: *P*=0.0564; and EXE versus CTR: *P*=0.0644); however, higher values of CTR versus FOS were observed using the Mann-Whitney *U* test, *P*=0.027.

In the BMI categorization analysis, the CTR showed the same level of distribution in the normal weight classification, 25%, a reduced level of distribution in the obesity classification, 25% to 16.67%, and an increased level of distribution in the overweight classification, 50% to 58.3%, between phases 1 and 4. The FOS had no changes in the distribution between phases 1 and 4, 54.54% in normal weight, 27.27% in overweight, and 18.19% in severe obesity. For the EXE, there was a reduction in the obesity classification, from 50% to 12.5%, and increased distribution in the overweight classification, from 25% to 62.5%, between phases 1 and 4.

Regarding the categorization of WHR, the CTR group showed a decrease in individuals in the low risk category, 41.66% to 25%, and an increase in the moderate risk category, 50% to 66.66%. The FOS group reduced the number of individuals in the high and very high risk categories to 0%, 18.19% and 9.09% to 0%, respectively. The CEO group showed an increase in the distribution of individuals in the high risk category from 37.5% to 50%.

The intragroup analysis was only made for BM, BMI, %FM, FFM, and WHR parameters. CTR intragroup data from phases 2 and 3 were not collected. In the intragroup analysis, FOS showed that %FM in phase 4 was significantly higher than that in phase 3 (*P*=0.0098), and FFM in phase 3 was significantly higher than that in phase 1 (*P*=0.0041). The EXE showed a statistical difference in BM, with phase 2 being significantly lower than phase 1 (*P*=0.0422), and, for BMI, phase 2 was significantly lower than phase 1 (*P*=0.0418).

### 4.2. Metabolic Profile

The results from TAG, CHOL, HDL, and LDL were verified in phases 1 and 4, and there was no significant difference between CTR, FOS, and EXE (Table 2).

### 4.3. Inflammatory Profile

**Figure 1:**
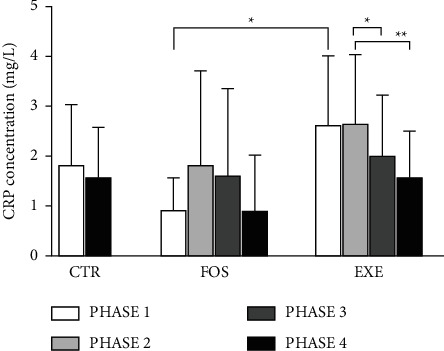
CRP concentration (mg/L) analysis in celiac disease patients and healthy people. CTR—healthy control group, *n* = 12; FOS—CD patients submitted to fish oil supplementation, *n* = 11; and EXE—CD patients submitted to fish oil supplementation and aerobic exercise, *n* = 8. ^*∗*^*P* < 0.05; ^*∗∗*^*P* < 0.01. CRP (ml/L)—C-reactive protein expressed in milligrams per liter.

In Figure 1, EXE versus FOS showed higher values of CRP in phase 1 (*P*=0.0216). The intragroup analysis of EXE showed that phases 3 and 4 were significantly lower than phase 2 (*P*=0.0427 and *P*=0.0091, respectively). There was no difference between phases 1 and 4 in CTR.

The test performed for IL-6 measurement had a sensitivity level of 1.5 pg/ml, and, in phase 1, 6 values of CTR, 3 values of FOS, and 3 values of EXE were below this sensitivity level. In phase 4, we also had data below sensitivity levels, 4 values of CTR, 3 values of FOS, and 6 values of EXE.

**Figure 2:**
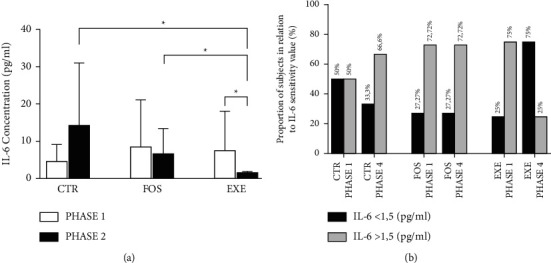
IL-6 results—inter- and intragroup analysis. (a) Groups distribution without IL-6 values <1.5 pg/ml: CTR—healthy control group, step 1, *n* = 6, step 4, *n* = 8; FOS—CD patients submitted to fish oil supplementation, phases 1 and 4, *n* = 8; and EXE—CD patients submitted to fish oil supplementation and aerobic exercise, phase 1, *n* = 6, step 4, *n* = 2; ^*∗*^*P* < 0.05. IL-6—interleukin-6 expressed in pictograms per milliliter. (b) CTR—healthy control group, *n* = 12; FOS—CD patients submitted to fish oil supplementation, *n* = 11; and EXE—CD patients submitted to fish oil supplementation and aerobic exercise, *n* = 8. Results are shown in percentage (%) of individuals.

In this context, Figure 2(a) shows the intergroup and intragroup analyses of IL-6 with the excluded values. In phase 1, no statistical difference was detected, CTR (7.58 ± 6.86 pg/ml) versus FOS (11.44 ± 14.24 pg/ml), *P*=0.9825, CTR (7.58 ± 6.86 pg/ml) versus EXE (9.48 ± 11.64 pg/ml), *P*=0.675, and FOS (11.44 ± 14.24 pg/ml) versus EXE (9.48 ± 11.64 pg/ml), *P*=0.7984. In step 4, higher values were observed in CTR (30.33 ± 29.71 pg/ml) versus FOS (8.95 ± 6.71 pg/ml), *P*=0.0401, and no statistical difference was detected in CTR (30.33 ± 29.71 pg/ml) versus EXE (8.65 ± 9.54 pg/ml), *P*=0.177, and FOS (8.95 ± 6.71 pg/ml) versus EXE (8.65 ± 9.54 pg/ml), *P*=0.722. The amount of below-sensitivity IL-6 values does not allow for the inclusion of EXE in the intragroup statistical tests. The intragroup analysis of CTR and FOS showed no statistical difference (*P*=0.1492 and *P*=0.8438, respectively).

Regarding the sensitivity limitation, Figure 2(b) shows the proportion of individuals with IL-6 more than or less than 1.5 pg/ml in a longitudinal analysis. Between phases 1 and 4, CTR showed an increase of 16.6% in the proportion of individuals with IL-6 >1.5 pg/ml, FOS showed no changes, and the proportion of individuals with IL-6 >1.5 pg/ml was reduced by 50% for the EXE group.

### 4.4. Average Caloric Intake

Average caloric intake was assessed at two phases, phases 1 and 4, for the FOS and EXE groups (Table 3). Comparing the groups in phase 4, we observed higher values of LIP in FOS versus EXE, *P*=0.0294.

### 4.5. Correlation Analysis

**Figure 3:**
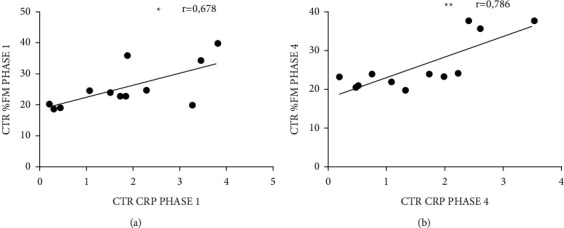
Correlation analysis. CTR—healthy control group, *n* = 12. ^*∗*^*P* < 0.05; ^*∗∗*^*P* < 0.01. CRP—C-reactive protein expressed in milligrams per liter (ml/L); %FM—fat mass expressed as a percentage.

Figure 3 shows the correlations of the longitudinal analysis. In phase 1, there was a positive correlation between CRP and %FM in the CTR (*P*=0.016) (Figure 3(a)). In phase 4, there was a positive correlation between CRP and %FM in the CTR (*P*=0.004) (Figure 3(b)).

## 5. Discussion

In the intergroup analysis of anthropometric parameters, we did not detect significant differences in BM, BMI, WHR, and %FM in any of the phases, a result that differs from the reviewed literature, which generally indicates that celiac disease patients with a gluten-free diet present lower values within these parameters [4]. For the BMI categorization, a previous study suggested a higher probability of CD patients, with or without a gluten-free diet, to be in the underweight classification [22]; however, our sample showed a smaller number of underweight CD patients, with prevalence in the normal weight classification.

Actually, the gluten-free diet, through a multifactorial benefit apparatus, is well established as a treatment that promotes an increase in BM and BMI in celiac disease patients [4], and this may contribute as an explanatory factor for the anthropometric parameters with similar values to healthy persons found in this study. However, in a cross-sectional analysis of this study, there was no correlation between the mean time of a gluten-free diet or adherence to a gluten-free diet with any anthropometric parameters (data not shown).

In the intragroup analysis of BMI, we observed that EXE showed a significant reduction in the obesity category, 50% to 12.5%. Considering that the FOS group does not exhibit changes in categorization and that previous studies indicate that fish oil has no effects on BM and BMI [23], it is suggested that the observed modulation is much more sensitive to regular physical exercise, mainly because of its ability to decrease BM [24].

Regarding %FM, the literature shows conflicting results, and our findings corroborate with studies that indicate equal values in these parameters between celiac disease patients and healthy people [4]. A possible explanation for this could be due to the sample eating habits and the inclusion criteria of this research. Nutritional recommendations from the Medicine Institute [25] consider macronutrient intake through estimated intake values, adequate intake values, and acceptable intake distribution ranges associated with the reduced risk of chronic disease. In comparison to the estimated and adequate values, our group had a higher CHO and PTN consumption in phases 1 and 4, and it was not possible to compare the LIP values. Considering the acceptable intake distribution ranges associated with the reduced risk of chronic disease, FOS and EXE presented values within the established references for CHO, PTN, and LIP. In the inclusion criteria of this study, a restriction towards people with moderate or high physical activity patterns was established, which inevitably caused the eligible people to become sedentary. Regarding the combination of factors, if average CHO and PTN consumption levels are higher than the recommendations associated with a history of physical inactivity, this may contribute to increased lipogenic activity, with a consequent increase in stored fat and increasing the %FM values.

In the intragroup analysis of the WHR, CTR increased the risk from the low to moderate category, while in the FOS group a reduction in the distribution of high and very high risk categories was observed. Unexpectedly, the EXE group recorded an increase within the very high risk category. Regarding these results, it appears that fish oil has an isolated ability to modulate fat accumulation in the waist and hip regions, which is in accordance with the meta-analysis of Schichun et al. [23].

FOS showed lower values of FFM than EXE and CTR in phase 1 but equal values in phase 4, suggesting an increase in these values during intervention. FOS also presented a significant increase in FFM in phase 3 and %FM in phase 4. Additionally, EXE showed a significant decrease in BM and BMI in phase 2. A previous study by Hill et al. [26] reported that physical exercise associated with fish oil supplementation significantly reduced %FM during a 12-week intervention, and the study by Noreen et al. [27] observed a significant increase in FFM through fish oil supplementation over a period of six weeks. Moreover, some studies suggest that fish oil consumption is related to FM reduction (kg) [27] and %FM [28]. Regarding these studies and our observations, the data suggests an increase in FFM through the isolated effects of fish oil supplementation. Additionally, the increase in %FM only in the FOS group and not in the EXE may be indicative of physical exercise, having the ability to suppress this increase, in addition to the higher consumption of LIP in the FOS group when compared to the EXE in phase 4.

There were no significant differences in the intergroup analysis regarding metabolic profile. When comparing American Heart Association (AHA) [29] references with Brazilian Society of Cardiology (SBC) [30] references, the TAG values of all the groups correspond to healthy people for AHA and SBC (<150 mg/dL). The CHOL values are within the range predicted by SBC (<190 mg/dL) and not by AHA (150 mg/dL). Regarding HDL, AHA, and SBC reference values, the FOS was within the range of the reference values (>50 mg/dL) in phases 1 and 4, and EXE was below the reference values in phase 1, but, in phase 4, it was within the range of reference values, and CTR was within the range of reference values in phase 1 but in phase 4 was below the reference values. The observed data suggests that, although not significant, EXE improved HDL regarding referential values. As our study does not include an intervention with a group performing only physical exercises, it is not possible to define what was the most important intervention in relation to HDL modulation. The LDL analysis is a bit more complex, once the AHA reference establishes an ideal value of 100 mg/L and SBC recommends a reference according to risk ranges. In our analysis, all groups were above the AHA reference in phases 1 and 4 and were in accordance with the SBC reference, only when the individuals were classified in the low risk range.

A decrease of CRP in EXE occurred gradually, and the reduction of phase 4 was more significant than phase 3, suggesting that a longer intervention time may be related to better results. Although FOS did not show a significant reduction in CRP, the behavior of the averages during the phases suggests an increase followed by stabilization, while the CTR average shows an increased level scenario. The results suggest that aerobic exercise in association with fish oil supplementation is more capable of modulating CRP when compared to only fish oil supplementation in celiac disease patients or the absence of interventions in healthy people.

The analysis of CRP in CTR demonstrated a positive correlation betwen CRP and %FM in phases 1 and 4. Indeed, there is a large dataset in the literature characterizing adipose tissue as an endocrine organ responsible for the production of molecules related to the inflammatory process. The study by Park et al. [31] of 100 adult individuals with no history of inflammatory disease or cancer suggests a significant relationship between CRP and IL-6 with anthropometric BMI, WHR, and visceral adipose tissue. Bo et al. [32] demonstrated significance (*P* ≤ 0.001) in the positive correlations between IL-6 versus CRP, IL-6 versus %FM, and CRP versus BM, BMI, and WHR. In addition, the study by Anty et al. [33] identified higher CRP expression in obese individuals and also greater CRP gene expression by the liver and adipose tissue.

Regarding the IL-6 analysis, in the studies by Manavalan et al. [5] and Tetzlaff et al. [6], celiac disease patients, with and without a gluten-free diet, showed higher IL-6 values than the healthy control group, a pattern that was not primarily observed in the analysis of our groups. This may have occurred because we had a reduction in the sample size due to the amount of values below the sensitivity level of the test. Even so, we observed significantly lower values in FOS versus CTR in phase 4. The decrease in the sample size due to the sensitivity of the test was also reported by Bautista et al. [34] when analyzing IL-6 (reduction of 15.4% of the sample) and TNF-*α* (reduction of 46.6% of the sample) in 196 people. It is important to note that the difficulty in recruiting participants and the amount of values below the sensitivity limit set a limitation for this study.

However, when analyzing EXE results in phases 1 and 4, we observed a 50% increase in the proportion of individuals with values <1.5 pg/ml, while CTR increased the number of individuals with IL-6 >1.5 pg/ml, and FOS did not show any changes. This data suggests that aerobic exercise associated with fish oil supplementation decreases IL-6 values. This reduction could not be statistically tested due to the reduction of the EXE sample size between phases 1 and 4, from 8 to 2 individuals, as a consequence of test sensitivity. In addition, IL-6 values below 1.5 pg/ml are related to the absence of a history of hypertension and hypercholesterolemia, diabetes, smoking, and a higher frequency of exercise [35].

Regarding the inflammatory profile response to fish oil supplementation, the results obtained in this study differ in part from the literature on decreases in IL-6 [14, 36] and CRP [14]. No intragroup reductions were detected within the respective parameters, and there was only a difference between CTR and FOS in IL-6 of phase 4. A possible answer may be the existence of a relationship between dosage and supplementation time; however, previous reviewed studies showed conflicting results, indicating better results with a higher dosage and time [13], with a similar dosage and lesser time [14], and with a lesser dosage and the same time [36]. Other factors that could have interfered in our results for the inflammatory profile were dietary limitations and the poor absorption of nutrients that characterize celiac disease patients [3]. However, some studies showed that CD patients, when compared to healthy people, had a similar dietary intake of EPA and DHA [37], equal serological concentration of EPA, and higher serological concentration of DHA [38, 39].

Thus, the data currently available in the literature suggests that the dosage of EPA and DHA, the supplementation time of the present study, and the pathophysiological characteristics of celiac disease do not necessarily characterize limiting factors for the manifestation of anti-inflammatory effects from fish oil.

## 6. Conclusions

The dataset obtained in this work differs from the literature regarding the morphophysiological and immunometabolic parameters in celiac disease patients. Fish oil supplementation alone did not promote changes in anthropometric and metabolic parameters; however, it promoted a statistical difference in IL-6. When fish oil supplementation was associated with aerobic exercise, it promoted a significant reduction in CRP and in the proportion of individuals with IL-6 >1.5 pg/ml, characterizing an intervention with corrective and preventive potential in relation to disorders associated with chronic inflammation.

The absence of a group submitted only to aerobic exercise does not allow us to discriminate between the biological effects that are dependent on the association between interventions and those exclusively dependent on aerobic exercise.

Studies are needed to clarify the impact of limiting factors on the experimental design of this research, such as (a) the qualitative and quantitative composition of experimental groups, which was composed by a reduced size and a female gender prevalence; (b) more precise inclusion criteria regarding the time since diagnosis of celiac disease; (c) complementary measurements of protocol efficiency; (d) tests with higher sensitivity for the detection of IL-6; and (e) complementary measurements of liver disease parameters that could be correlated to CD.

## Figures and Tables

**Table 1:** Age and anthropometric parameters in celiac disease patients and healthy people—intergroup analysis.

Phase	1			2		3		4		
Group	CTR	FOS	EXE	FOS	EXE	FOS	EXE	CTR	FOS	EXE
*N*	12	11	8	11	8	11	8	12	11	8
Age (years)	31.92 ± 4.9	36.5 ± 10.7	38.2 ± 8.8					31.92 ± 4.9	36.5 ± 10.7	38.2 ± 8.8
Height (m)	1.71 ± 0.07^1^	1.62 ± 0.05^1^	1.59 ± 0.04					1.71 ± 0.07^2^	1.62 ± 0.05^2^	1.59 ± 0.04
BM (kg)	78.3 ± 14.3	67.5 ± 16.7	75.9 ± 18.5^6^	67.6 ± 16.7	74.9 ± 18.5^6^	69.2 ± 16.6	68.7 ± 9.7	77.8 ± 14.1	67.9 ± 19.3	74.1 ± 17.1
BMI (kg/m^2^)	26.6 ± 3.5	25.6 ± 5.7	28.2 ± 4.4^7^	25.6 ± 5.7	28 ± 4.3^7^	26.3 ± 5.6	27.7 ± 3.9	26.4 ± 3.4	23.4 ± 10.2	27.7 ± 3.5
BMI normal (%)	25	54.54	25					25	54.54	25
BMI overweight (%)	50	27.27	25					58.33	27.27	62.5
BMI obesity (%)	25	0	50					16.67	0	12.5
BMI severe obesity (%)	0	18.19	0					0	18.19	0
%FM	25.5 ± 7.1	28.9 ± 9.3	34.3 ± 5.6	28.8 ± 10.3	33.7 ± 4.85	27.5 ± 10.5^4^	32.7 ± 4.1	26.1 ± 6.7	28.3 ± 10.8^4^	33.5 ± 4.3
FFM (kg)	58.3 ± 12.5^3^	46.4 ± 5.9^3,5^	49.3 ± 13.9^3^	46.6 ± 5.7	45.1 ± 4.3	47.3 ± 6.5^5^	45.3 ± 4.2	57.5 ± 12	46.6 ± 6.9	49.3 ± 12.8
WHR	0.78 ± 0.09	0.77 ± 0.06	0.80 ± 0.09	0.75 ± 0.07	0.77 ± 0.05	0.72 ± 0.,05	0.79 ± 0.09	0.79 ± 0.08	0.73 ± 0.04	0.80 ± 0.08
WHR low (%)	41.66	27.27	12.5					25	36.36	12.5
WHR moderate (%)	50	45.45	37.5					66.66	63.64	25
WHR high (%)	8.34	18.19	37.5					8.34	0	50
WHR very high (%)	0	9.09	12.5					0	0	12.5

^1,2,3,4,5,6,7^Equal numerical symbols indicate significant difference from each other: ^1^ANOVA, *P*=0.0061; ^2^ ANOVA, *P*=0.0061; ^3^ Kruskal-Wallis, *P*=0.0221, not significant in Dunn's posttest but significant to CTR versus FOS in Mann-Whitney *U* test, *P*=0.027; ^4^pairing *t*-test, *P*=0.0098; ^5^pairing *t*-test, *P*=0.0041; ^6^pairing *t*-test, *P*=0.0422; ^7^pairing *t*-test, *P*=0.0418. CTR—healthy control group, *n* = 12; FOS—CD patients submitted to fish oil supplementation, *n* = 11; and EXE—CD patients submitted to fish oil supplementation and aerobic exercise, *n* = 8. Age in years; height in meters; BM—body mass in kilograms; BMI—body mass index; %FM—fat mass percentage; FFM—fat-free mass in kilograms; WHR—waist-to-hip ratio. Subjects were classified into BMI and WHR categories, with results shown as a percentage.

**Table 2:** Metabolic profile in celiac disease patients and healthy people—intergroup analysis.

Phase	1			4		
Group	CTR	FOS	EXE	CTR	FOS	EXE
TAG	94.9 ± 43.7	102.1 ± 47.5	142.6 ± 99.8	78.9 ± 30.9	91.8 ± 52.8	113.4 ± 43.5
CHOL	176.9 ± 45.4	171.3 ± 35.7	179.4 ± 45.4	167.3 ± 59.9	163.4 ± 53.0	171.4 ± 21.5
HDL	50.1 ± 11.3	56.9 ± 13.2	46.2 ± 9.6	45.42 ± 10.9	54.8 ± 10.4	53.8 ± 13.1
LDL	126.8 ± 47.8	114.4 ± 35.1	133.2 ± 40.15	121.9 ± 50.90	108.6 ± 22.6	117.6 ± 14.1

CTR—healthy control group, *n* = 12; FOS—CD patients submitted to fish oil supplementation, *n* = 11; and EXE—CD patients submitted to fish oil supplementation and aerobic exercise, *n* = 8. TAG—triglycerides, CHOL—cholesterol, HDL—high-density lipoprotein and LDL—low-density lipoprotein expressed in milligrams per deciliter.

**Table 3:** Average caloric intake analysis.

Phases	1		4	
Group	FOS	EXE	FOS	EXE
CAL (kcal)	1661 ± 391.8	1605 ± 476.1	1547 ± 328	1259 ± 240.1
PTN (g/d)	68.5 ± 19.2	72.9 ± 26.4	67.6 ± 23.1	69.0 ± 19.1
CHO (g/d)	207.3 ± 83.2	184.3 ± 93.5	178.9 ± 38.7	174.5 ± 76.9
LIP (g/d)	62.4 ± 23.1	71.5 ± 28.9	59.2 ± 17.0^1^	4.5 ± 10.9^1^

^1^Equal numerical symbols indicate a significant difference between each other: ^1^*t*-test, *P*=0.0294. FOS—CD patients submitted to fish oil supplementation, *n* = 11; EXE—CD patients submitted to fish oil supplementation and aerobic exercise, *n* = 8. CAL—calories expressed in kilocalories (kcal); PTN—proteins, CHO—carbohydrates; LIP—lipids expressed in grams per day (g/d).

## Data Availability

The data used to support the findings of this study are available from the corresponding author upon request.

## Abbreviations

%FM: Fat mass
*ω*-3: Omega-3
ALA: *α*-Linolenic acid
BM: Body mass
BMI: Body mass index
CHOL: Cholesterol
CAL: Calories
CDAT: Celiac dietary adherence test
CTR: Control group of healthy people
DHA: Docosaexaenoic acid
EPA: Eicosapentaenoic acid
EXE: Celiac disease patients submitted to fish oil supplementation and aerobic exercise
FFM: Fat-free mass
FOS: Celiac disease patients submitted to fish oil supplementation
HDL: High-density lipoproteins
HLA: Human leukocyte antigen
IL: Interleukin
INF-*γ*: Interferon-gamma
LDL: Low-density lipoproteins
LIP: Lipids
PTN: Proteins
TAG: Triglycerides
TNF-*α*: Tumor necrosis factor-alpha
WHR: Waist-to-hip ratio.
